# Synergistic Enhancement of Electron Dynamics and Optical Properties in Zeolitic Imidazolate Framework-8-Derived Zinc Oxide via Surface Plasmon Resonance Effects of Silver Nanoparticles under UV Irradiation

**DOI:** 10.3390/ma17133193

**Published:** 2024-06-29

**Authors:** Jaewon Lee, Byoung-Nam Park

**Affiliations:** Department of Materials Science and Engineering, Hongik University, 72-1 Sangsu-dong, Mapo-gu, Seoul 04066, Republic of Korea; leejaewon1108@gmail.com

**Keywords:** ZIF-8, surface plasmonic resonance, silver nanoparticles

## Abstract

This study investigates the surface plasmon resonance (SPR)-induced UV photoresponse of zinc oxide (ZnO) derived from zeolitic imidazolate framework-8 (ZIF-8) to assess the influence of silver nanoparticles (Ag NPs) on the photoresponse behavior of metal–organic framework (MOF)-derived ZnO. The initial synthesis involved a thermal treatment in air to convert ZIF-8 into ZnO. We noted enhanced optical absorption both in the UV and visible spectra with the deposition of Ag NPs onto the ZIF-8-derived ZnO. Additionally, the presence of Ag NPs in the ZnO resulted in a substantial increase in current, even without any light exposure. This increase is attributed to the transfer of electrons from the Ag NPs to the ZnO. Photocurrent measurements under UV illumination revealed that the photocurrent with Ag NPs was significantly higher—by two orders of magnitude—compared with that without Ag NPs. This demonstrates that SPR-induced absorption markedly boosted the photocurrent, although the current rise and decay time constants remained comparable to those observed with ZnO alone. Although Ag NPs contribute electrons to ZnO, creating a “pre-doping” effect that heightens baseline conductivity (even in the absence of light), this does not necessarily alter the recombination dynamics of the photogenerated carriers, as indicated by the similar rise and decay time constants. The electron transfer from Ag to ZnO increases the density of charge carriers but does not significantly influence their recombination.

## 1. Introduction

The intersection of nanoparticle (NP) engineering and photocatalysis presents an exciting frontier for the development of advanced materials capable of harnessing solar energy for environmental and energy applications [1,2,3]. Among the various materials being explored, zinc oxide (ZnO) derived from zeolitic imidazolate framework-8 (ZIF-8) integrated with silver nanoparticles (Ag NPs) stands out due to its potential in enhancing ultraviolet (UV) photoresponses through surface plasmon resonance (SPR) [4,5,6,7]. ZIF-8 is a type of metal–organic framework (MOF) distinguished by its unique structure, merging the characteristics of both zeolites and traditional MOFs. It consists of zinc (Zn) ions, which act as the metal nodes, connected by imidazolate linkers derived from imidazole, a nitrogen-containing heterocyclic compound. ZIF-8 features a highly porous architecture with large internal cavities and small pore apertures. This high porosity enables ZIF-8 to adsorb and store a wide range of molecules, making it valuable for applications such as gas storage and separation [8,9].

Further, during thermal annealing, the carbon from the ZIF-8 linkers can be incorporated into the ZnO lattice as dopants. Carbon doping can modify the electronic properties of ZnO, potentially enhancing its photocatalytic activity and other properties. Depending on the synthesis conditions, carbon may form as a coating on the ZnO particles or as part of a composite structure, influencing the photocatalytic properties of ZnO.

The integration of Ag NPs with ZnO derived from ZIF-8 leverages the unique properties of both components. ZnO is a well-known photocatalyst with strong UV light absorption capabilities, while Ag NPs are renowned for their plasmonic properties, which can manipulate light to enhance a material’s photocatalytic efficiency [10,11,12]. The synergy between ZnO and Ag NPs via SPR not only extends the light absorption from the UV into the visible spectrum but also facilitates the improved separation and transportation of photogenerated electron-hole pairs [13,14]. This is critical in maximizing the use of solar light, particularly the abundant UV component. Most of all, the interaction between plasmonic Ag NPs and ZnO can lead to a significant improvement in photocatalytic activity. The enhanced photoresponse under UV light is crucial for accelerating reactions that degrade pollutants, purify water, and break down organic compounds, which are vital for environmental sustainability [15,16].

In a previous study, Ag-doped ZnO derived from a ZIF-8 precursor through adsorption and sintering methods demonstrated superior photocatalytic activity with an enhanced absorption into the red wavelength region. Its photocatalytic efficiency significantly improved from 92.32% to 99.64%. Moreover, Ag/ZnO showed robust stability, maintaining 97.48% efficiency after four degradation cycles, making it highly effective for degrading organic dyes [17]. Abdi et al. reported that a porous Ag-doped ZIF-8 nanocomposite was synthesized and used as a photocatalyst for degrading various cationic and anionic dyes under visible light [18]. The photocatalytic performance of Ag-doped ZIF-8 surpassed that of a simple mixture of ZIF-8 and AgNO_3_ due to the heterojunction between them.

The rationale for previous studies is multifaceted, encompassing the desire to enhance photocatalytic processes, improve solar energy conversion, and refine detection methods across various scientific disciplines. ZIF-8-derived ZnO already possesses remarkable photocatalytic properties, and the integration of Ag NPs is hypothesized to induce SPR, which is expected to further augment its response to UV light. The SPR phenomenon is anticipated to extend the absorption spectrum, potentially allowing for the exploitation of the visible light range in addition to the UV range, thus harnessing a broader swath of the solar spectrum.

From an environmental perspective, as mentioned above, the augmented photoresponse is vital for the photodegradation of persistent pollutants, offering a pathway to clean energy initiatives and sustainable environmental remediation [19,20]. In the commercial sector, enhanced-UV-response materials can lead to the development of new products, from UV-protective coatings to advanced sensors with heightened sensitivity. Therefore, the study of ZnO derived from ZIF-8 combined with Ag NPs under UV illumination transcends basic scientific inquiry—it is an endeavor that could catalyze advancements across a spectrum of real-world applications.

This study is designed to uncover the essential mechanisms that lead to an improved UV photoresponse, focusing on the interaction between Ag nanoparticles and ZnO that facilitates charge transfer, as well as the amplification of optical absorption brought about by SPR. At the forefront of this exploration is the study of ZnO derived from ZIF-8, particularly when interfaced with plasmonic Ag NPs under UV illumination. The integration of Ag NPs can significantly enhance the photocatalytic performance of ZnO. The SPR effect of Ag NPs helps to harness more light energy, particularly from the UV spectrum, which is crucial for accelerating photocatalytic reactions.

However, research focusing specifically on the enhancement of the SPR effects induced by Ag NPs in ZIF-8-derived ZnO under UV light is relatively limited. While ZIF-8 is widely studied for its gas storage and separation capabilities, its conversion to ZnO for photocatalytic applications is a more recent development [21,22]. The use of plasmonic NPs like Ag NPs to enhance the optical properties of materials is a growing field, but its application in conjunction with ZIF-8-derived ZnO under UV light is less explored. While there are extensive studies on photocatalysis, the specific scenario involving Ag-NP-enhanced ZIF-8-derived ZnO under UV light involves complex interactions that have not been extensively documented.

This paper aims to dissect the reasons behind the intensified interest in the photoresponse of ZIF-8-derived ZnO when synergized with plasmonic Ag NPs under the influence of UV light. We probe the charge transfer between Ag NPs and ZIF-8-derived ZnO in the dark and correlate how Ag NPs enhance the UV photoresponse of ZnO derived from ZIF-8 through SPR.

## 2. Materials and Methods

### 2.1. Synthesis of ZIF-8

Zinc nitrate hexahydrate (Sigma, St. Louis, MO, USA, Zn(NO_3_)_2_·6H_2_O, 10 mmol) was used as the source of zinc. 2-methylimidazole (Hmim, 40 mmol) served as the organic linker. Both reagents were dissolved in methanol, depending on the desired solubility and reaction conditions. A solution of zinc nitrate hexahydrate was prepared by dissolving it in methanol to create a clear solution. Separately, 2-methylimidazole was dissolved in methanol at a concentration adjusted to maintain a molar ratio of 1:4 relative to the zinc source. The 2-methylimidazole solution was gradually added to the zinc solution without stirring to initiate the synthesis of ZIF-8. The mixture was then left to stand at room temperature for crystallization to occur over 1 day. Following crystallization, the ZIF-8 crystals were separated from the mother liquor by centrifugation (8000 rpm for 10 min). This step was followed by multiple washes with methanol to remove unreacted starting materials and any by-products. The washed crystals were dried under vacuum at 120 °C to eliminate any residual solvents, thereby stabilizing the crystal structure for storage and further analysis. For the conversion of Zn to ZnO, the samples underwent a thermal treatment at 500 °C in an air atmosphere for a duration of 5 h.

### 2.2. Structural and Electrical Characterizations of ZIF-8 and ZIF-8-Derived ZnO

The characterization of the synthesized ZIF-8 crystals was performed using X-ray diffraction (XRD) to verify their crystallinity and phase purity. The crystal morphology was examined using scanning electron microscopy (SEM, Hitachi S-3200H, Tokyo, Japan). The optical absorption properties of the ZnO derived from ZIF-8 were determined with a spectrophotometer (UV-670). The crystal morphology was examined using scanning electron microscopy (SEM). The optical absorption properties of the ZnO derived from ZIF-8 were determined with a spectrophotometer. Bottom-contact interdigitated devices were fabricated by patterning drain and source electrodes, composed of Au/Ti, onto a 200 nm thick SiO_2_ gate dielectric layer using standard photolithography techniques. This critical step established the electrical pathways for the device. The gate electrode was formed using a highly doped silicon substrate, chosen for its high conductivity and optimal compatibility with the SiO_2_ dielectric, with a resistivity between 0.001 and 0.002 Ωcm. The electrical properties of the ZIF-8-derived ZnO devices post thermal oxidation were characterized under UV illumination.

For optical absorption and electrical measurements, a 5 mg/mL ZIF-8 solution in ethanol was spin-coated onto a glass substrate at 2000 rpm for 30 s and then subjected to annealing. Subsequently, Ag NPs were thermally evaporated onto the bottom-contact interdigitated device at a rate of 1 Å/s within a vacuum chamber maintained at approximately 10^−6^ torr. The electrical properties of the ZIF-8-derived ZnO devices post thermal oxidation were characterized under UV illumination with a UV-light-emitting diode lamp (365 nm). Connections were established to the source, drain, and gate electrodes to enable electrical measurements using a semiconductor parameter analyzer (HP4145B).

## 3. Results and Discussion

**Figure 1:**
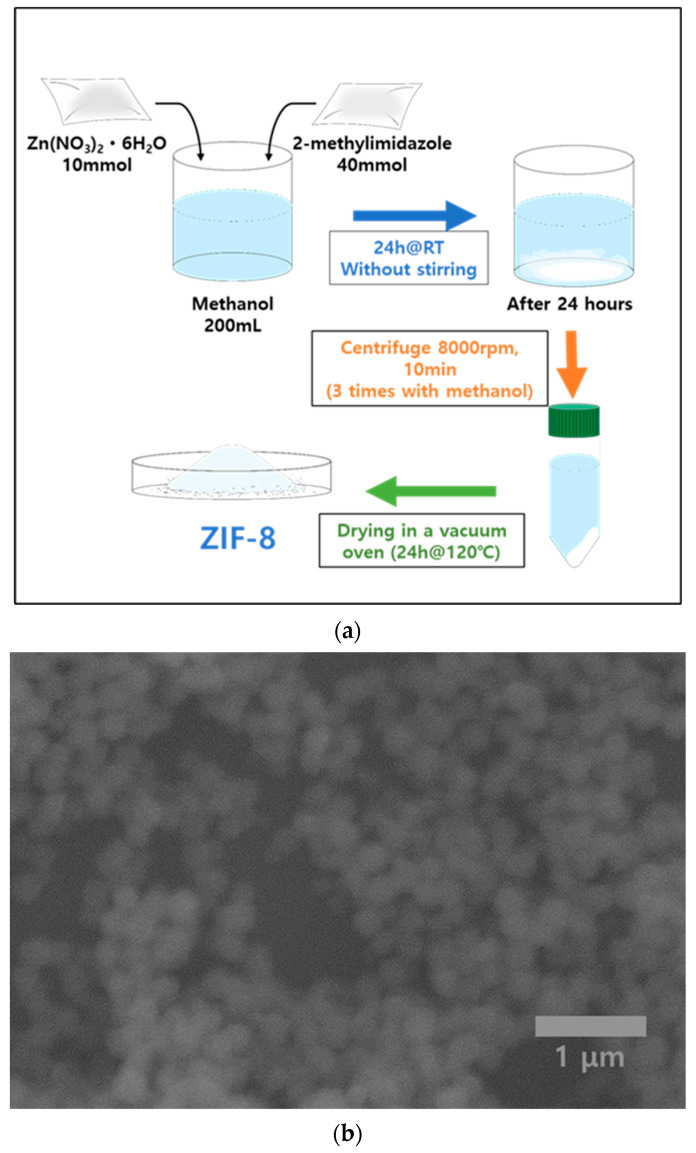
(**a**) Diagram illustrating the process of synthesizing ZIF-8. (**b**) SEM image showcasing the produced ZIF-8 crystals.

Figure 1a presents the detailed synthesis process of ZnO derived from ZIF-8, beginning with the initial production of ZIF-8 and followed by a critical thermal oxidation step. The SEM image shown in Figure 1b captures the complex and textured surface of the synthesized ZIF-8. The image reveals a dense array of nanoscale features that are indicative of the highly crystalline nature of ZIF-8. Typically, ZIF-8 crystallizes in polyhedral or spherical shapes, and the image clearly shows these forms, along with a relatively uniform distribution of particles or crystallites across the examined surface. Although there is visible agglomeration, it does not obscure the distinctness of individual particles, each of which contributes to the overall texture of the material.

Moreover, the scale of these features, predominantly within the micrometer range, is consistent with what is expected for ZIF-8 crystals synthesized under controlled conditions. This uniformity in size and shape is essential for applications that rely on consistent material properties, such as catalysis or adsorption, where surface area and porosity play critical roles. The image thus not only demonstrates the successful synthesis of ZIF-8 but also underscores the material’s suitability for further conversion into functionally enhanced ZnO through the subsequent thermal treatment.

**Figure 2:**
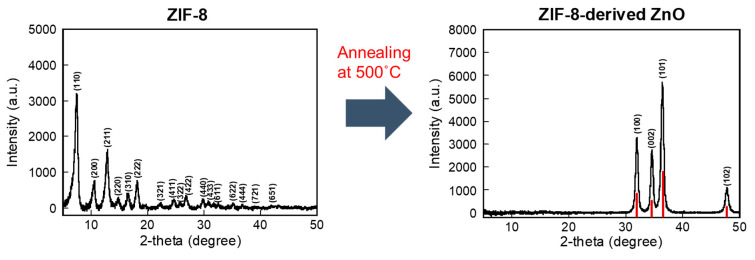
X-ray diffraction patterns comparing ZIF-8 before and after thermal annealing to produce ZIF-8-derived ZnO. The red bars represent theoretical XRD peaks.

In Figure 2, the X-ray diffraction (XRD) pattern for activated ZIF-8 shows sharp, distinct peaks, reflecting its crystalline structure and emphasizing its high crystallinity and typical porous nature. These peak positions and intensities are characteristic of the specific atomic arrangement within the ZIF-8 framework, matching its recognized crystal structure. Following annealing at 500 °C for 5 h in air, which involves oxidation, the XRD pattern undergoes a significant transformation indicative of the conversion from ZIF-8 to ZnO. The original sharp peaks of ZIF-8 disappear, replaced by new peaks indicative of the hexagonal wurtzite structure of ZnO. This change in the diffraction pattern distinctly demonstrates the transformation from the hybrid organic–inorganic ZIF-8 to a purely inorganic ZnO framework, unveiling a fresh array of crystallographic planes and interplanar distances.

**Figure 3:**
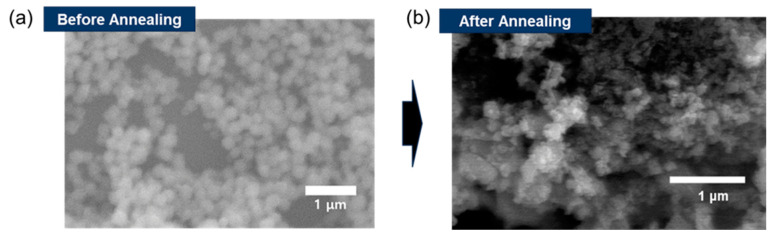
SEM comparison images of (**a**) original ZIF-8 and (**b**) ZIF-8-derived ZnO.

Based on the SEM images provided in Figure 3, the transformation of ZIF-8 before and after annealing can be described through a comparison of the observed morphological changes. Before annealing in Figure 3a, the SEM image shows the ZIF-8 with a homogenous and highly textured surface consisting of numerous nanoscale features. The structure appears as a closely packed array of fine particles or crystallites with a size of ~250 nm. The pre-annealed ZIF-8 features are likely to reflect the intrinsic polyhedral shapes commonly associated with crystalline ZIF-8. The particles exhibit a level of uniformity with little to no evidence of coalescence or fusion, suggesting a well-controlled synthesis process. Post annealing, the SEM image in Figure 3b reveals significant morphological changes. The surface texture becomes more pronounced with increased roughness, likely due to the thermal decomposition of the organic linkers in ZIF-8 and the formation of ZnO. The annealed structure seems less uniform with more pronounced features, which can indicate the conversion of ZIF-8 to ZnO particles that may possess different sizes and shapes due to the thermal process. The granularity of the material appears more distinct, with the increased visibility of individual particles or aggregates, suggesting that annealing leads to the growth and possible agglomeration of ZnO particles.

**Figure 4:**
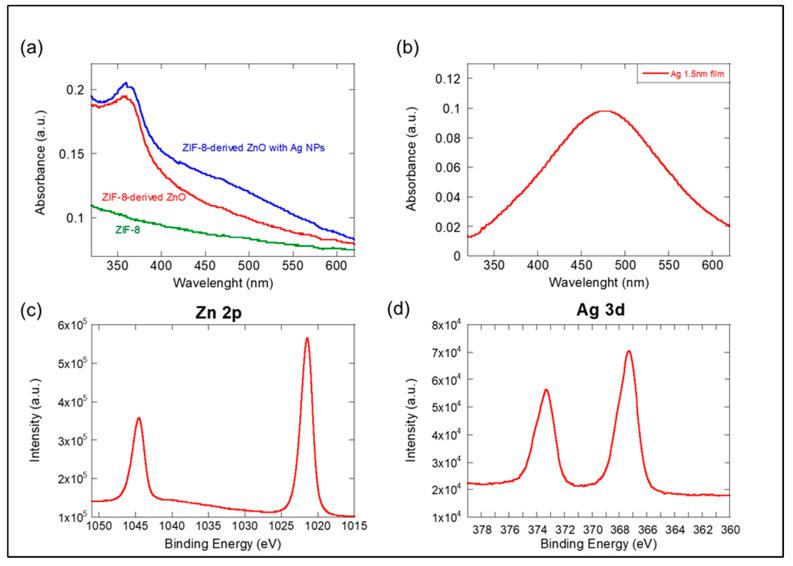
Optical absorption spectra of (**a**) ZIF-8, ZIF-8-derived ZnO, and ZIF-8-derived ZnO with Ag NPs; (**b**) optical absorption of Ag NPs on a glass substrate. XPS spectra of Ag/ZIF-8-derived ZnO for (**c**) Zn 2p and (**d**) Ag 3d.

The modification of the optical properties following the deposition of Ag NPs onto ZIF-8-derived ZnO is a significant phenomenon, as shown in Figure 4a,b. The absorption data present the absorption characteristics of ZIF-8 in various stages of modification and the isolated absorption behavior of Ag NPs. The green curve in Figure 4a represents the absorption of pristine ZIF-8, which typically exhibits a low absorption across the visible range due to its inherent transparent nature as an MOF. The red curve indicates the absorption post thermal annealing at 500 °C for 5 h in air, which results in ZIF-8-derived ZnO. The thermal treatment significantly alters the absorption characteristics due to the conversion from the organic–inorganic hybrid ZIF-8 to the inorganic ZnO, which inherently has a higher absorption in the UV region. The blue curve shows the absorption of ZIF-8-derived ZnO after the deposition of Ag NPs. The introduction of Ag NPs enhances the absorption across the spectrum, particularly in the regions corresponding to the plasmonic resonances of the Ag NPs. This indicates the Ag NPs are contributing to the absorption profile due to SPR effects, which enhance the interaction with light, thereby increasing the material’s overall absorption. The peak observed is the characteristic SPR band of Ag, as shown in Figure 4b, which is typically in the visible range. The presence of this peak confirms the plasmonic nature of the Ag NPs.

When comparing the spectra, the effect of incorporating Ag NPs on the photo-absorptive properties of ZIF-8-derived ZnO becomes clear. The Ag NPs not only introduce a new absorption peak due to their SPR but also enhance the overall absorption capacity of the ZnO. This could potentially translate into improved photocatalytic activity, as a higher absorption of light energy allows for the more efficient generation of electron-hole pairs, which are crucial for photocatalysis. The deposition of Ag NPs onto ZIF-8-derived ZnO substantially modifies the material’s optical absorption capabilities, an observation that is crucial for developing highly efficient, plasmonically-active photocatalytic materials.

The XPS spectra in Figure 4c,d verify the chemical composition and elemental valent state at the Ag/ZIF-8-derived ZnO heterojunction interface.

The doublet peaks observed at approximately 1021.6 eV and 1044.7 eV in Figure 4c result from spin-orbit coupling, a typical feature in XPS spectra for zinc. The 23.1 eV separation between these peaks is characteristic of Zn^2+^ in ZnO, confirming the presence of zinc in the +2 oxidation state. Similarly, the doublet peaks at around 368 eV and 374 eV in Figure 4d are separated by about 6 eV, which is typical for silver and confirms the presence of metallic Ag.

**Figure 5:**
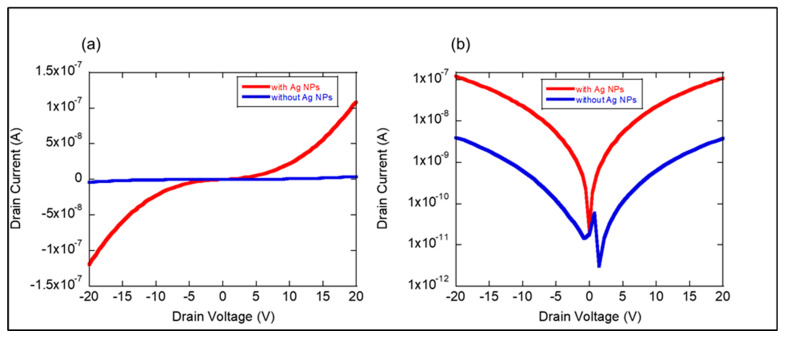
Current–voltage characteristics of ZIF-8-derived ZnO with and without Ag NPs, presented on (**a**) linear and (**b**) logarithmic scales in the absence of light.

Importantly, the formation of the ZnO/Ag NP interface led to an increase in dark current compared with the ZnO without Ag NPs, as seen in Figure 5. The increase in dark current for the ZnO with deposited Ag NPs, when compared with the ZnO without Ag NPs, can be attributed to several factors related to the electronic interactions at the ZnO/Ag interface. Ag NPs have a lower work function compared with ZnO. When they come into contact, electrons will naturally transfer from Ag to ZnO until their Fermi levels align. This transfer of electrons introduces additional charge carriers in the ZnO, increasing its electrical conductivity even without illumination (dark conditions) [23,24]. Ag NPs might modify the surface states of ZnO by passivating or filling surface traps. These surface states typically trap charge carriers, but the presence of Ag could saturate these traps, freeing up more carriers to contribute to the dark current [25].

**Figure 6:**
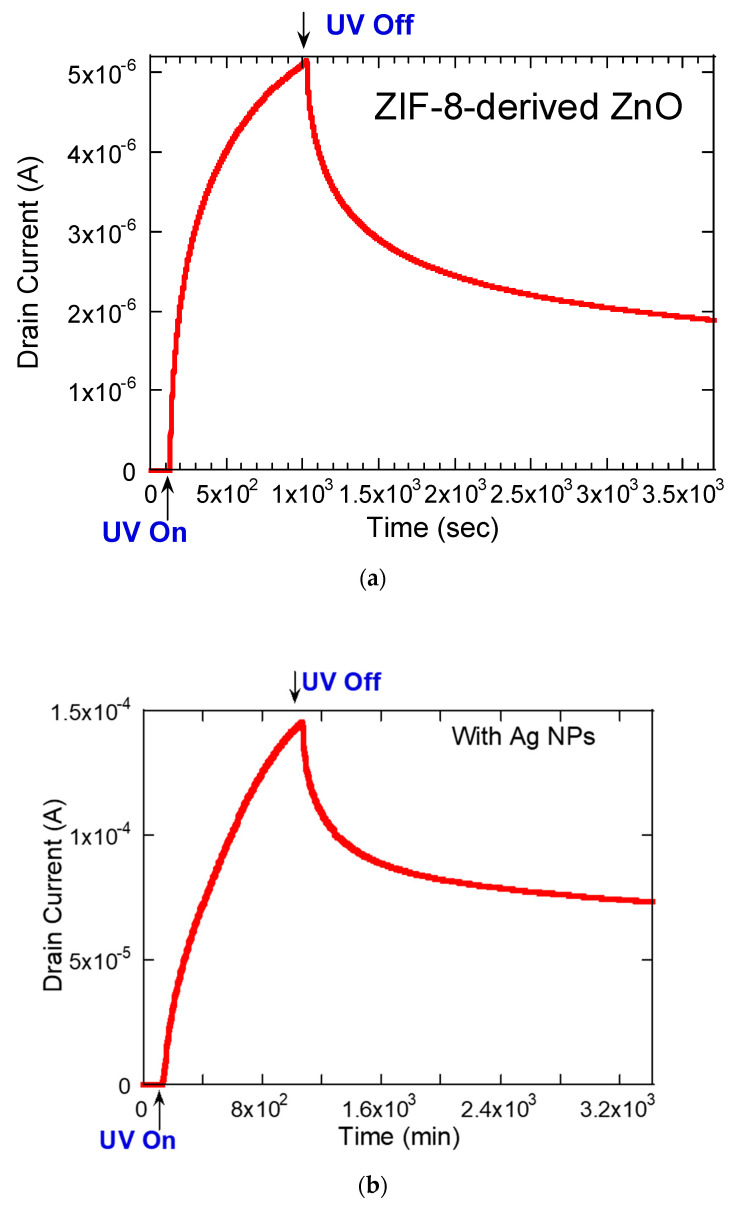

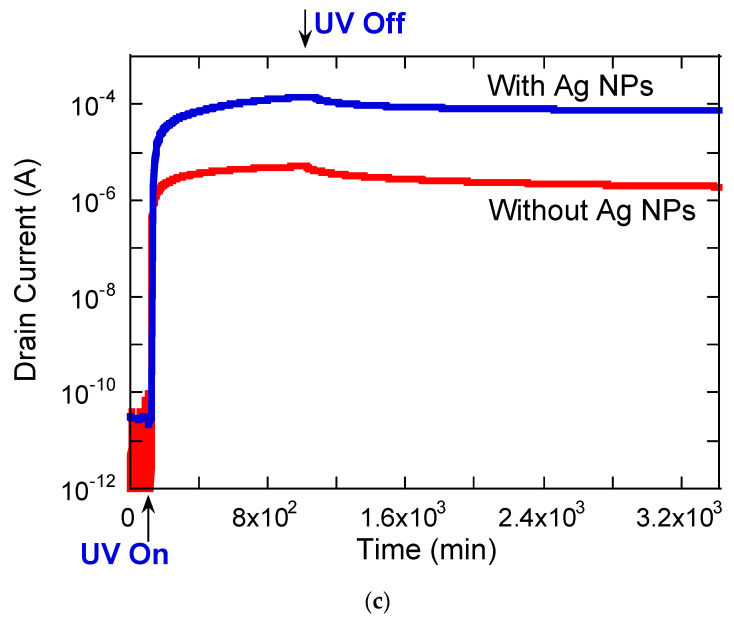
Current–time response measurements of ZIF-8-derived ZnO (**a**) without and (**b**) with Ag NPs under cycles of UV light exposure and non-exposure, with the drain voltage held constant at 0.05 V. (**c**) Logarithmic comparison of plots (**a**,**b**).

The photoresponse to UV light of ZIF-8-derived ZnO was probed using time-resolved current measurements in Figure 6. When subjected to UV radiation, the ZnO conductivity dramatically escalated, showing an increase in current over five orders of magnitude compared with its non-illuminated state, as shown in Figure 6a. Once the UV source was turned off, there was a steady decay in the current. Notably, the boost in conductivity due to UV exposure was thirty times higher in the presence of Ag NPs than in their absence, as compared in Figure 6b,c. This observed modulation in current, both upon the application and withdrawal of UV light, can be linked to substantial fluctuations in the concentration of free electrons, which are contingent upon the cycles of oxygen adsorption and desorption on the ZnO surface [26,27,28].

Under UV light exposure, the electrical conductivity of ZnO markedly increases, primarily due to the surge in mobile charge carriers. This change is caused by the shift in surface energy bands, or band bending, which is initiated by the detachment of oxygen molecules from the ZnO surface. Typically, these oxygen molecules attach to surface sites of ZnO, acting as electron traps and accepting electrons to become negatively charged adsorbed oxygen ions (O_2_^−^(*ad*)), as captured by the reaction O_2_(*g*) + *e^−^* → O_2_^−^(*ad*). This electron capture creates a deficit of free electrons in the depletion layer, reducing its electrical conductivity. Consequently, the energy bands at the surface bend upwards. Yet, when UV light is introduced, it generates electron-hole pairs, and the photogenerated holes prompt the recombination with O_2_^−^(*ad*), dislodging the oxygen ions from the surface. This leads to a prompt increase in the number of free electrons within the depletion layer as the bound oxygen ions are released.

The non-instantaneous increase in current upon UV illumination and the subsequent non-instantaneous decrease when the UV is turned off can be explained by the dynamics of oxygen adsorption and desorption on the surface of ZnO, as well as the subsequent changes in the charge carrier concentrations [26,29]. The interaction of oxygen molecules with the ZnO surface involves charge transfer processes that are not instantaneous. When oxygen molecules are adsorbed, they capture free electrons from the ZnO, creating a negatively charged adsorbed state (O_2_^−^(*ad*)) and leaving behind a depleted layer with fewer free carriers. The reverse process, where photogenerated holes recombine with O_2_^−^(*ad*), liberates electrons back into the conduction band. These interactions involve diffusion and trapping dynamics that are time-dependent. Under UV illumination, electron-hole pairs are generated within the ZnO. The time it takes for these pairs to separate and for the holes to migrate to the surface and recombine with adsorbed O_2_^−^(*ad*) is finite. The separation of charge carriers is influenced by the material’s morphology and crystallinity, as well as the presence of defects or impurities. The rate of the adsorption and desorption of oxygen on the ZnO surface is also limited by the mass transport of oxygen molecules to and from the surface. Even when photogenerated holes are available to recombine with the adsorbed oxygen, the physical process of oxygen molecule desorption from the surface takes time.

The kinetics of adsorption–desorption and charge carrier dynamics are governed by various physical processes that occur on different time scales, from femtoseconds to milliseconds or even longer. These processes are not instantaneous, and therefore the current changes observed during UV illumination and post-illumination periods are gradual rather than immediate.

The analysis of current decay with and without Ag NPs, as shown in Figure 6a and Figure 6b, respectively, was modeled using the second-order exponential function *I*(*t*) = *I*(∞) + *I*_1_exp(−*t*/*τ*_1_) + *I*_2_exp(−*t*/*τ*_2_), where *I*(∞) is the steady state current. This model elucidated two distinct timescales for electron release, represented by *τ*_1_ and *τ*_2_, which indicate the presence of various electron trapping states within the ZnO. Specifically, the analysis identified two separate decay processes featuring shorter and longer lifetimes—111 s and 625 s with Ag NPs and 100 s and 769 s without Ag NPs, respectively. These similar lifetimes suggest that while Ag NPs enhance conductivity, they do not significantly alter the rate of carrier recombination. The decay time constants and their corresponding parameters are detailed in Table 1.

**Figure 7:**
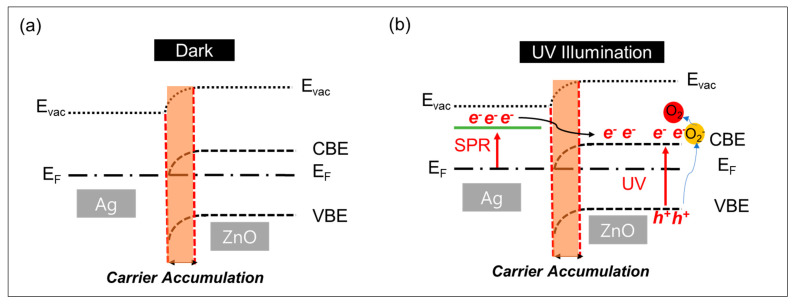
Energy band diagrams illustrating the interface between ZIF-8-derived ZnO and Ag both in (**a**) darkness and (**b**) under UV light exposure.

The considerable increase in current observed in the dark when Ag NPs are present and the even more dramatic increase under UV illumination can be attributed to the mechanisms depicted in Figure 7a for the dark condition and Figure 7b for the UV condition. For the Ag/ZnO system, ZnO functions as an *n*-type semiconductor with a work function of close to the conduction band edge (5.3 eV) and a band gap of about 3.3 eV [30]. Its conduction band edge (CBE) and valence band edge (VBE) are positioned at −5.3 eV and −8.6 eV, respectively. Ag NPs have a work function of 4.26 eV [31]. When Ag contacts ZnO, the lower work function of Ag leads to electron transfer from Ag to ZnO until their Fermi levels equalize. This electron transfer initiates an accumulation layer where electrons concentrate in the space charge region, as shown in Figure 7a, substantially boosting the current [32].

Under UV illumination, the excitation of Ag prompts electrons to move from Ag NPs to the CBE of ZnO. This is facilitated because the surface plasmonic state of the Ag NPs is positioned above the CBE of ZnO, as illustrated in Figure 7b. Additionally, the holes created from band-to-band transitions in ZnO under UV exposure recombine with electrons that are bound to oxygen molecules on the surface. The recombination process releases mobile electrons resulting from the desorption of oxygen from the surface, thereby causing a significant rise in the current, as illustrated in Figure 7b.

## 4. Summary and Conclusions

This research paper explores the importance of examining the surface plasmon resonance (SPR)-induced UV photoresponse of ZnO derived from ZIF-8 enhanced with Ag NPs. It highlights the transformative potential of this hybrid material for applications in photocatalysis and more. The study delves into how Ag NPs augment the UV photoresponse of ZnO derived from ZIF-8, specifically through the mechanism of SPR. The process begins by thermally converting ZIF-8 to ZnO, after which Ag NPs are added, resulting in increased optical absorption and electrical current even without UV light due to electron transfer from Ag to ZnO. Under UV exposure, ZnO with Ag NPs shows a photocurrent two orders of magnitude higher than ZnO alone, confirming that SPR significantly amplifies the photoresponse. Despite this, the timing of current changes remains similar, suggesting that while Ag NPs boost conductivity, they do not affect the rate of carrier recombination.

This strategy can be utilized for the photocatalytic degradation of pollutants in water and air, photoelectrochemical cells, and the inactivation of pathogens through enhanced photocatalytic activity. To elucidate the mechanisms of electron transfer and SPR enhancement at the nanoscale, advanced spectroscopic techniques (such as time-resolved photoluminescence spectroscopy), along with theoretical modeling, are necessary. By investigating the SPR-induced UV photoresponse of ZnO derived from ZIF-8 with Ag NPs, this research contributes to the broader field of material science and nanotechnology, paving the way for the development of more efficient, cost-effective, and environmentally friendly photocatalytic systems.

## Figures and Tables

**Table 1:** Summary of shorter (*τ*_1_) and longer (*τ*_2_) lifetimes and fitting parameters of the bi-exponential current decay.

	*I_D_*(∞) (A)	*τ*_1_ (s)	*τ*_2_ (s)	*I*_1_ (A)	*I*_2_ (A)	*R^2^*
Without Ag NPs	1.919 × 10^−6^	100	769	0.052	6.454 × 10^−6^	0.9997
With Ag NPs	7.520 × 10^−5^	111	625	0.449	1.760 × 10^−4^	0.9997

## Data Availability

Data are contained within the article.
